# A real-world study of treatment patterns among patients with osteoporotic fracture: analysis of a Japanese hospital database

**DOI:** 10.1007/s11657-022-01201-x

**Published:** 2023-01-23

**Authors:** Hiroshi Hagino, Yoko Yoshinaga, Etsuro Hamaya, Tzu-Chieh Lin, Mayank Ajmera, Juliana Meyers

**Affiliations:** 1 School of Health Sciences, Tottori University, Tottori, Japan; 2 Medical Affairs, Amgen K.K, Tokyo, Japan; 3 Center for Observational Research, Amgen, Inc, Thousand Oaks, USA; 4 Department of Health Economics and Outcomes Strategy, RTI Health Solutions, Research Triangle Park, NC USA

**Keywords:** Osteoporosis, Osteoporosis fracture, Treatment patterns, Antiosteoporotic medications, Retrospective database analysis, Treatment adherence

## Abstract

*****Summary***:**

Health records of patients hospitalized for osteoporotic fracture were analyzed. Prior to the index hospital admission, most patients were not receiving any antiosteoporotic treatment. During the index hospitalization visit, 25.5% of patients received antiosteoporotic treatment. The most common treatment regimens were active vitamin D_3_, bisphosphonates, and teriparatide.

**Purpose:**

To examine the real-world treatment patterns and factors associated with receipt of treatment among Japanese patients with osteoporotic fracture.

**Methods:**

We retrospectively analyzed health records of patients who were hospitalized for osteoporotic fracture between February 2016 and February 2018 in Japan. The type and duration of treatment with antiosteoporotic medications prescribed during hospital stays and after discharge were examined using descriptive statistics. Demographic and clinical factors (e.g., age, previous diagnoses, Charlson Comorbidity Index scores) associated with osteoporotic treatment were explored using multivariable logistic regression.

**Results:**

A total of 112,275 patient medical records were evaluated, including 56,574 records from patients with hip fracture, 26,681 records from patients with vertebrae fracture, and 29,020 patients with non-vertebral non-hip fractures. Prior to the index hospital admission, most patients (91.7%, *n* = 102,919) were not receiving any antiosteoporotic treatment. For those receiving treatment, active vitamin D_3_ (51.1%, *n* = 4778) and bisphosphonates (47.5%, *n* = 4441) were the most common. During the index hospitalization visit, 25.5% (*n* = 28,678) of patients received treatment for their fracture, including active vitamin D_3_ (*n* = 17,074), bisphosphonates (*n* = 10,007), and teriparatide (*n* = 4561). Upon discharge, 41.5% (*n* = 46,536) of patients returned to their home and 34.3% (*n* = 38,542) of patients were transferred to a different hospital or medical care facility. Variables associated with receipt of treatment at follow-up included older age, previous diagnoses of osteoporosis and fracture, and higher Charlson Comorbidity Index scores.

**Conclusion:**

Despite osteoporotic fracture being a major health concern within older Japanese populations, treatment with antiosteoporotic medication regimens remains generally low.

**Supplementary Information:**

The online version contains supplementary material available at 10.1007/s11657-022-01201-x.

## Introduction

Osteoporosis and osteoporotic fracture are major health concerns for the estimated 15 million people in Japan with osteoporosis [1], with greater lifetime risk for fractures to the spine and hip as individuals increase in age [2, 3]. Osteoporotic fracture has been associated with an increased mortality risk of between 10 and 20% in the 1st year after the fracture, and among individuals with osteoporotic fracture, functional status has been found to decrease by approximately 60% when compared with prefracture functional status levels [1]. Therefore, it is important to consider pharmacological treatments as preventative measures among patients with diagnosed osteoporosis and adequately treat osteoporotic fracture after it has occurred [4].

Clinical guidelines in Japan recommend pharmacological treatment for individuals with a history of osteoporosis and osteoporotic fracture [5]. Despite this recommendation, previous studies have observed that only 20% of individuals in Japan with osteoporosis received antiosteoporotic treatment [1]. Additional reports have indicated that only 18.7% of patients were prescribed antiosteoporotic treatment within 1 year of follow-up for a hip fracture [6]. More recent studies have examined individuals who are at greater risk for fractures, including women who are perimenopausal or postmenopausal and who have an osteoporosis diagnosis and individuals who have comorbid conditions such as cancer. These studies have found that some patients are unwilling to take medication, while other patients might not be actively receiving care for their osteoporosis; furthermore, the risk for osteoporotic fracture can increase in patients who are not given pharmacological treatment for osteoporosis [7–9]. However, additional studies assessing real-world data are needed to further understand the treatment patterns of antiosteoporotic therapy following hospitalization for an osteoporotic fracture.

To address these knowledge gaps, this study was conducted to describe the recent treatment landscape among patients in Japan with osteoporotic fracture. We retrospectively analyzed health records of Japanese patients who were hospitalized for osteoporotic fracture to examine the type and duration of treatment with antiosteoporotic agents prescribed to patients during hospital stays and after hospital discharge. Other demographic variables and clinical characteristics, including sex, age, fracture location, previous diagnosis of osteoporosis, and comorbid health conditions, also were examined to further understand their association with treatment patterns.

## Methods

### Study design

The study design was a retrospective database analysis of patients diagnosed and hospitalized for osteoporotic fracture. Patient medical records and administrative data were obtained from the Medical Data Vision (MDV) database in Japan. The MDV is a large database composed of a wide range of administrative and claims data for more than 30 million patients across 400 hospitals [10]. MDV primarily contains data from hospitals that use a claims-based payment system, referred to as the Diagnosis Procedure Combination (DPC) system, that is administrated by the Japanese Ministry of Health, Labor, and Welfare. As of December 2018, 18% of DPC hospitals in Japan were included in the MDV database [11]. The database contains information such as sex, birth year, department of care, dates of medical encounters, payment plans, diagnoses and medical procedures (defined by *International Classification of Diseases, 10th Revision* [ICD-10] codes), laboratory orders and results, and prescriptions associated with inpatient and outpatient hospital encounters [10]. This study was reviewed by the Public Health Research Foundation Review Board and was determined to be exempt from human research according to the Ethical Guidelines for Medical and Health Research Involving Human Subjects in Japan.

### Study population and timeline

All data were collected from the MDV database between February 1, 2015 and February 28, 2019 (Online Resource 1, Figure S-1). The study analytic cohort was comprised of patients who were at least 50 years old and had been admitted to the hospital with a diagnosis of osteoporotic fracture (the index hospitalization date was defined as the date that a patient was initially admitted to the hospital for his/her fracture and extended to the date of discharge). Eligible patients needed to have at least one visit (documented in their medical record) to the hospital occurring at least 12 months before the index hospitalization (12-month period before the date of their index hospitalization termed baseline period), and at least one visit to the hospital occurring at least 12 months after the date of discharge from the index hospitalization (12-month period after the date of discharge from the index hospitalization termed follow-up period). Accordingly, to allow for the baseline and follow-up periods, the study cohort included patients who were admitted to the hospital with a diagnosis of osteoporotic fracture between February 1, 2016 and February 28, 2018 (Online Resource 1, Figure S-2).

### Demographic and clinical characteristics

The primary demographic variables that were documented included sex, age, and the skeletal site of the osteoporotic fracture at the index hospitalization date. Patient data were divided into three mutually exclusive cohorts based on the skeletal site of fracture observed during the index hospitalization: hip, vertebrae (spine), and non-vertebral non-hip (NVNH). NVNH fracture included fractures to the rib(s), sacrum, pubis, upper end of the humerus, lower or upper end of the radius or ulna, forearm, femur, or upper end of the tibia but excluded fractures to the skull, phalanges, and toes. Consistent with previously published literature [12–14], patients in all fracture cohorts were selected based on diagnoses using ICD-10 codes (Online Resource 1, Table S-1). Patients with multiple fracture sites during the index hospitalization were excluded from the study. Clinical variables documented during the baseline period included the presence of comorbid health conditions as described by the Charlson Comorbidity Index (CCI) [15]. The CCI includes 20 categories of comorbidities, as defined by diagnosis codes, with associated weights corresponding to the severity of the comorbid condition of interest [15]. Other documented comorbid conditions (e.g., cerebrovascular disease, congestive heart failure, peripheral vascular disease, and myocardial infarction) were identified by ICD-10 codes and/or by laboratory results.

The variables documented at the index hospitalization date included a previous diagnosis of osteoporosis and/or a previous diagnosis of osteoporotic fracture; smoking index as reported in the MDV database (reported as the number of cigarettes smoked per day multiplied by the number of years of smoking); activities of daily living (ADL) scores as reported in the MDV database, including information on eating, transferring, grooming, use of the toilet/going to the toilet, bathing, level ground walking, stairs, and clothing; route (hospitalized from home, transferred from another hospital, or admitted from another healthcare facility) of admission to the hospital; and the type of hospitalization (planned visit, emergency, or unplanned nonemergency). The clinical variables documented during follow-up included the discharge destination (home, another hospital or healthcare facility, or a geriatric care center) from the hospital, health outcome status (healing/curing of fracture, remission, no change, exacerbation, death, or other) upon discharge, and the presence of home care (care within the same hospital, care within a different hospital, no care, or unknown care status) after discharge.

### Treatment patterns

Among the available data, the rates and the class of pharmacological treatment that patients received during the baseline period, index hospitalization, and follow-up were documented. Across these three time points, pharmacological treatment data included the number and percentage of patients receiving an antiosteoporotic agent, as well as the average number of treatment days. Treatment data during the baseline period included the use of antiosteoporotic agents, the class of antiosteoporotic agents received, and the average number of days from the time of treatment initiation to the index hospitalization date. Treatment data during the index hospitalization also included the classes of antiosteoporotic agents received, the number of days from the index hospital admission date to the date on which patients received the first observed antiosteoporotic agent, and the average number of days patients received medications while in the hospital. Finally, treatment data during the follow-up period included the classes of antiosteoporotic agents received, time from hospital discharge to posthospital treatment initiation, the first observed antiosteoporotic agent treatment regimen after discharge (treatment must be received within 14 days after discharge to be included), and the number of patients who either discontinued, switched/restarted, or augmented their treatment regimen.

Treatment discontinuation was defined as the point at which patients had a minimum refill gap (approximately 60 days) between their most recent observed prescription and the end of the 12-month follow-up period. Switching of treatment was defined as the observation of an alternative medication without a refill of the original medication and was based on the observation of prescription fills for other medication classes following the discontinuation date of the original medication. Augmentation was defined as the initiation of a new medication class with continued use of the original medication for a minimum of 60 days of combined use. Adherence to the treatment regimen was also measured using the medication possession ratio (MPR). The MPR was calculated as follows: the sum of the days’ supply of medication in an observation period divided by the number of days in the observation period. Categories of MPR (e.g., deciles) were also documented and a dichotomous indicator of adherence was computed. Based on the MPR threshold of 0.8 commonly used to define medication adherence in the osteoporosis literature [16–18], a value greater than or equal to 0.80 was considered adherent and a value less than 0.80 was considered nonadherent. Adherence to the first observed treatment regimen was measured by patients’ cumulative exposure to medication during the follow-up period.

### Statistical analyses

All statistical analyses were conducted using SAS Version 9.4 (SAS Institute; Cary, North Carolina). A descriptive analysis of continuous study variables generated mean values, medians, and standard deviations (SDs) from all available data. Categorical study variables were analyzed with frequency distributions and reported with numbers and percentages. Multivariable logistic regression models were used to examine the associations between patient demographic and clinical characteristics and receipt of treatment, and the outcomes of the associations were presented as odds ratios with 95% confidence intervals (CIs). Independent variables, which included age, sex, CCI score, previous osteoporosis diagnosis, previous osteoporotic fracture diagnosis, discharge destination, and medication use at discharge, were included as covariates in the regression models.

## Results

### Demographic and clinical characteristics

A total of 112,275 patient medical records were evaluated, including 56,574 records from patients with hip fracture, 26,681 records from patients with vertebrae fracture, and 29,020 of patients with NVNH fractures. The average age of the total patient sample was 80.0 years (SD, 10.5) (Table 1). The average age was 83.1 years (SD, 9.3) for patients with hip fracture, 80.2 years (SD, 9.1) for patients with vertebrae fracture, and 73.8 years (SD, 11.1) for patients with NVNH fractures. Of the total patient sample, 74.8% (*n* = 83,950) were female; 51.8% (*n* = 43,453) of women had hip fractures, 21.7% (*n* = 18, 221) of women had vertebrae fractures, and 26.5% (*n* = 22, 276) of women had NVNH fractures. The mean CCI score during the baseline period for the total sample was 1.1 (SD, 2.3). The mean CCI score was 1.0 (SD, 2.1) among patients with NVNH fracture, 1.0 (SD, 2.2) among patients with hip fracture, and 1.4 (SD, 2.5) among patients with vertebrae fracture.

**Table 1:** Patient demographics and clinical characteristics

Characteristic	Overall (*N* = 112,275)	Hip fracture (*n* = 56,574)	Vertebrae fracture (*n* = 26,681)	NVNH fracture (*n* = 29,020)
Age at index hospital visit, *y*				
Mean (SD), median	80.0 (10.5), 82.0	83.1 (9.3), 85.0	80.2 (9.1), 82.0	73.8 (11.1), 74.0
Range (min, max)^a^	50–100	50–100	50–100	50–100
Age category, *n* (%), *y*				
50–59	5,632 (5.0)	1,319 (2.3)	833 (3.1)	3,480 (12.0).)
60–69	14,106 (12.6)	4,212 (7.5)	2,719 (10.2)	7,175 (24.7)
70–79	25,856 (23.0)	10,296 (18.2)	7,164 (26.9)	8,396 (28.9)
80–89	46,643 (41.5)	26,372 (46.6)	12,320 (46.2)	7,951 (27.4)
≥ 90	20,038 (17.9)	14,375 (25.4)	3,645 (13.7)	2,018 (7.0)
Sex, *n* (%)				
Male	28,325 (25.2)	13,121 (23.2)	8,460 (31.7)	6,744 (23.2)
Female	83,950 (74.8)	43,453 (76.8)	18,221 (68.3)	22,276 (76.8)
Length of hospital stay, d				
Mean (SD), median	32.2 (28.9), 24	36.5 (28.5), 28.0	35.2 (29.5), 27.0	21.3 (26.1), 12.0
Range (min, max)	1–878	1–656	1–878	1–834
Previous osteoporosis diagnosis, *n* (%)				
No	105,976 (94.4)	54,757 (96.8)	24,088 (90.3)	27,131 (93.5)
Yes	6,299 (5.6)	1,817 (3.2)	2,593 (9.7)	1,889 (6.5)
Previous osteoporotic fracture diagnosis, *n* (%)				
No	111,471 (99.3)	56,396 (99.7)	26,237 (98.3)	28,838 (99.4)
Yes	804 (0.7)	178 (0.3)	444 (1.7)	182 (0.6)
CCI score in the baseline period				
Mean (SD), median	1.1 (2.3), 0.0	1.0 (2.2), 0.0	1.4 (2.5), 0.0	1.0 (2.1), 0.0
Range (min, max)	0, 22	0, 22	0, 22	0, 22
Smoking index^b^				
Mean (SD), median	104.5 (334.8), 0.0	91.0 (319.9), 0.0	131.0 (379.9), 0.0	106.4 (317.3), 0.0
Range (min, max)	0–9600	0–9000	0–9270	0–9600
Route of hospitalization, *n* (%)				
Hospitalized from home	92,303 (82.2)	40,437 (71.5)	24,556 (92.0)	27,310 (94.1)
Transferred from another hospital	8362 (7.5)	6153 (10.9)	1321 (5.0)	888 (3.1)
Transferred from a different ward in the same hospital	125 (0.1)	63 (0.1)	33 (0.1)	29 (0.1)
Hospitalized from a nursing/welfare facility	11,073 (9.9)	9686 (17.1)	708 (2.7)	679 (2.3)
Other	375 (0.3)	217 (0.4)	56 (0.2)	102 (0.4)
Type of hospitalization for index visit, *n* (%)				
Planned hospitalization	20,703 (18.4)	4553 (8.1)	3386 (12.7)	12,764 (44.0)
Planned rehospitalization	24 (0.0)	9 (0.0)	7 (0.0)	8 (0.0)
Unplanned hospitalization, nonemergency	40,677 (36.2)	17,292 (30.6)	14,575 (54.6)	8810 (30.4)
Emergency hospitalization	50,654 (45.1)	34,612 (61.2)	8662 (32.5)	7380 (25.4)

*Max*, maximum; *min*, minimum; *NVNH*, non-vertebral non-hip; *SD*, standard deviation

^a^Age > 100 was set by default to 100 years

^b^Reported as the number of cigarettes smoked per day times years of smoking, with 0 indicating no smoking history

At the index hospitalization visit, most patients had not been previously diagnosed with osteoporosis (94.4%, *n* = 105,976) and had not previously experienced an osteoporotic fracture (99.3%, *n* = 111,471). Furthermore, most patients (91.7%, *n* = 102,919) had not previously received treatment with antiosteoporotic medications. The majority of patients arrived at the hospital for an unplanned emergency visit (45.1%, *n* = 50,654) or an unplanned nonemergency visit (36.2%, *n* = 40,677); 18.4% (*n* = 20,703) of patients had planned their hospital visit. Overall, the most common comorbid health conditions observed at the index hospitalization visit were hypertension (39.0%, *n* = 43,795), dementia (14.9%, *n* = 16,691), diabetes without complications (14.0%, *n* = 15,667), cerebral vascular disease (11.8%, *n* = 13,290), and congestive heart failure (10.0%, *n* = 11,278). The proportions of incident diagnoses for common comorbidities at the index hospitalization were 9.3% (*n* = 10,452) for cerebrovascular disease, 7.5% (*n* = 8454) for congestive heart failure, 1.4% (*n* = 1616) for peripheral vascular disease, and 1.3% (*n* = 1510) for myocardial infraction (Online Resource 1, Table S-2).

Patients’ ability to engage in ADLs was assessed during their hospitalization (Table 2), with 88.6 to 97.8% of patients having data available across the different ADL categories. For patients with hip fracture, grooming, using the toilet, walking at ground level or on stairs, and bathing were the ADLs that required the most assistance: 60.1% (*n* = 33,974) needed assistance with grooming, 33.8% (*n* = 19,094) needed significant help with using the toilet, 39.5% (*n* = 22,362) needed significant help with ground-level walking, 52.2% (*n* = 29,555) were unable to walk stairs, and 78.0% (*n* = 44,130) were unable to bathe independently. Most patients with a fracture to the vertebrae (61.3%, *n* = 16,343) were also unable to bathe independently, and 31.5% (*n* = 8414) were unable to walk stairs. Similarly, 42.8% (*n* = 12,430) of patients with NVNH fractures were unable to bathe independently.

**Table 2:** Activities of daily living scores during index hospital visit

Activity	Overall, *n* (%)	Hip fracture, *n* (%)	Vertebrae fracture, *n* (%)	NVNH fracture, *n* (%)
Eating				
2 = able to cut up food, spread butter, etc. without help	68,674 (61.2)	28,328 (50.1)	18,267 (68.5)	22,079 (76.1)
1 = needs some help cutting or spreading	33,017 (29.4)	20,521 (36.3)	6568 (24.6)	5928 (20.4)
0 = needs to be fed	8101 (7.2)	6180 (10.9)	1240 (4.7)	681 (2.4)
Unknown/missing	2483 (2.2)	1545 (2.7)	606 (2.3)	332 (1.1)
Transferring				
3 = needs no help	51,315 (45.7)	15,925 (28.2)	13,923 (52.2)	21,467 (74.0)
2 = needs minor help	36,635 (32.6)	23,176 (41.0)	8228 (30.8)	5231 (18.0)
1 = needs major help but can sit unaided	8561 (7.6)	6201 (11.0)	1513 (5.7)	847 (2.9)
0 = cannot sit; needs skilled lift by 2 people	13,309 (11.9)	9751 (17.2)	2405 (9.0)	1153 (4.0)
Unknown/missing	2455 (2.2)	1521 (2.7)	612 (2.3)	322 (1.1)
Grooming				
1 = independent	58,020 (51.7)	20,970 (37.1)	15,773 (59.1)	21,277 (73.3)
0 = needs help	51,613 (46.0)	33,974 (60.1)	10,266 (38.5)	7373 (25.4)
Unknown/missing	2642 (2.4)	1630 (2.9)	642 (2.4)	370 (1.3)
Use of toilet/going to toilet				
2 = able to use toilet without physical or verbal help	54,061 (48.2)	17,523 (30.1)	14,645 (54.9)	21,893 (75.4)
1 = needs some help	30,121 (26.8)	18,369 (32.5)	7070 (26.5)	4682 (16.1)
0 = needs significant help	25,537 (22.8)	19,094 (33.8)	4343 (16.3)	2100 (7.2)
Unknown/missing	2556 (2.3)	1588 (2.8)	623 (2.3)	345 (1.2)
Bathing				
1 = able to bathe independently	34,765 (31.0)	9916 (17.5)	9269 (34.7)	15,580 (53.7)
0 = unable to bathe independently	72,903 (64.9)	44,130 (78.0)	16,343 (61.3)	12,430 (42.8)
Unknown/missing	4607 (4.1)	2528 (4.5)	1069 (4.0)	1010 (3.5)
Level ground walking				
3 = may use aid	45,969 (40.9)	12,917 (22.8)	12,481 (46.8)	20,571 (70.9)
2 = needs help of 1 person, verbal or physical, including help standing up	21,273 (19.0)	11,441 (20.2)	6164 (23.1)	3668 (12.6)
1 = independent in wheelchair, including able to negotiate doors and corners	9725 (8.7)	6492 (11.5)	1751 (6.6)	1482 (5.1)
0 = needs more help than described above	30,276 (27.0)	22,362 (39.5)	5214 (19.5)	2700 (9.3)
Unknown/missing	5032 (4.5)	3362 (5.9)	1071 (4.0)	599 (2.1)
Stairs				
2 = independent up and down, and can carry any necessary walking aid	35,462 (31.6)	8461 (15.0)	8804 (33.0)	18,197 (62.7)
1 = needs help, verbal or physical, or help carrying aid	21,343 (19.0)	10,834 (19.2)	6348 (23.8)	4161 (14.3)
0 = unable	42,612 (38.0)	29,555 (52.2)	8414 (31.5)	4643 (16.0)
Unknown/missing	12,858 (11.5)	7724 (13.7)	3115 (11.7)	2019 (7.0)
Clothing				
2 = independent putting on clothes, including fastening buttons, zips, etc	43,962 (39.2)	14,316 (25.3)	12,298 (46.1)	17,348 (59.8)
1 = needs some help, but can do at least half	36,874 (32.8)	19,477 (34.4)	8713 (32.7)	8684 (29.9)
0 = needs more help than this	28,849 (25.7)	21,163 (37.4)	5041 (18.9)	2645 (9.1)
Unknown/missing	2590 (2.3)	1618 (2.9)	629 (2.4)	343 (1.2)

*NVNH*, non-vertebral non-hip

### Treatment patterns

#### Baseline

Prior to the index hospital admission, most patients (91.7%, *n* = 102,919) were not receiving any antiosteoporotic treatment. Among the 9356 patients receiving treatment, active vitamin D_3_ (51.1%, *n* = 4778) and bisphosphonates (47.5%, *n* = 4441) were the most common (Table 4). Among the patients who were taking medication prior to the index hospital visit, the average number of days from the start of treatment to the index hospital visit was approximately 253 days (median, 312 days) across all cohorts.

#### Index hospital visit

The average length of stay during the index hospital visit was 32.2 days (median, 24 days), and most patients (82.2%, *n* = 92,303) were transferred to the hospital from their home (Table 1). During the index hospitalization visit, 25.5% (*n* = 28,678) of patients received treatment for their fracture (Table 3). Treatment initiation during the index hospitalization began a mean 9.8 days (median, 4 days) after the index hospital visit, and this inpatient treatment continued for a mean 27.5 days (median, 18 days) (Table 3). The most common types of medications given were active vitamin D_3_ (59.5%, *n* = 17,074), bisphosphonates (34.9%, *n* = 10,007), and teriparatide (15.9%, *n* = 4,561) (Table 4). Among patients receiving active vitamin D_3_ during their index hospitalization (*n* = 17,074), 21.7% (*n* = 3712) received combination treatment with a bisphosphonate, 3.4% (*n* = 585) received combination treatment with calcium, 3.7% (*n* = 631) received combination treatment with calcitonin, and 4.0% (*n* = 682) received combination treatment with a teriparatide. Calcitonin (9.4%, *n* = 2706) and selective estrogen receptor modulators (SERMs, [4.9%, *n* = 1401]) were also administered, and estrogen treatment was given to a small number of individuals (0.6%, *n* = 160).

**Table 3:** Treatment rates and adherence

Characteristic	Overall	Hip fracture	Vertebrae fracture	NVNH fracture
Treatment before the index hospital visit, *n* (%)				
No	102,919 (91.7%)	53,142 (93.9%)	22,933 (86.0%)	26,844 (92.5%)
Yes	9356 (8.3%)	3432 (6.1%)	3748 (14.1%)	2176 (7.5%)
No. of days from start of treatment to index hospital admission				
Mean (SD), median	252.7 (119.1), 312.0	256.0 (110.3), 308.0	242.0 (130.0), 311.0	265.9 (110.8), 317.0
Range (min, max)	1–365	1–365	1–365	1–365
No. of days in the baseline period with medication available				
Mean (SD), median	125.3 (140.8), 49.0	118.1 (138.0), 45.0	124.5 (141.9), 48.0	137.8 (142.7), 55.0
Range (min, max)	1–365	1–365	1–365	1–365
Treatment during the index hospital visit, *n* (%)				
No	83,597 (74.5%)	43,422 (76.8%)	15,882 (59.5%)	24,293 (83.7%)
Yes	28,678 (25.5%)	13,152 (23.3%)	10,799 (40.5%)	4727 (16.3%)
No. of days from admission to inpatient treatment initiation				
Mean (SD), median	9.8 (14.8), 4.0	11.4 (15.5), 7.0	8.2 (13.7), 2.0	8.6 (14.3), 3.0
Range (min, max)	0–230	0–196	0–162	0–230
No. of inpatient days with medication received				
Mean (SD), median	27.5 (29.3), 18.0	28.2 (29.5), 20.0	27.5 (29.5), 17.0	25.7 (28.6), 14.0
Range (min, max)	1–300	1–300	1–280	1–212
Treatment after index hospital visit, *n* (%)				
No	88,616 (78.9%)	47,183 (83.4%)	17,441 (65.4%)	23,992 (82.7%)
Yes	23,659 (21.1%)	9391 (16.6%)	9240 (34.6%)	5028 (17.3%)
No. of days from discharge to treatment initiation				
Mean (SD), median	17.2 (50.0), 1.0	16.0 (49.2), 1.0	22.1 (52.1), 1.0	15.1 (49.4), 1.0
Range (min, max)	1–365	1–365	1–365	1–365
Discharge destination, *n* (%)				
Transferred to another ward at the same hospital	57 (0.1%)	35 (0.1%)	14 (0.1%)	8 (0.0%)
Discharged home—check-ups done in the same hospital	46,536 (41.5%)	13,321 (23.6%)	13,192 (49.4%)	20,023 (69.0%)
Discharged home—check-ups held in another hospital	11,084 (9.9%)	4306 (7.6%)	3632 (13.6%)	3146 (10.8%)
Transferred to a different hospital or medical facility	38,542 (34.3%)	27,379 (48.4%)	7014 (26.3%)	4149 (14.3%)
Entry to geriatric health services facility	4585 (4.1%)	3155 (5.6%)	966 (3.6%)	464 (1.6%)
Entry to nursing care home	3064 (2.7%)	2590 (4.6%)	264 (1.0%)	210 (0.7%)
Entry to social welfare facility or private residential home	5232 (4.7%)	3996 (7.1%)	736 (2.8%)	500 (1.7%)
Died	1916 (1.7%)	1306 (2.3%)	455 (1.7%)	155 (0.5%)
Nursing medical institution	0 (0.0%)	0 (0.0%)	0 (0.0%)	0 (0.0%)
Other	1165 (1.0%)	445 (0.8%)	375 (1.4%)	345 (1.2%)
Outcome at the time of discharge, n (%)				
Healing/cured	104,552 (93.1%)	52,080 (92.1%)	24,597 (92.2%)	27,875 (96.1%)
Remission	352 (0.3%)	153 (0.3%)	137 (0.5%)	62 (0.2%)
No change	3079 (2.7%)	1757 (3.1%)	777 (2.9%)	545 (1.9%)
Exacerbation	97 (0.1%)	50 (0.1%)	34 (0.1%)	13 (0.0%)
Death	1901 (1.7%)	1301 (2.3%)	449 (1.7%)	151 (0.5%)
Other	2204 (2.0%)	1197 (2.1%)	651 (2.4%)	356 (1.2%)
After discharge home care, n (%)				
None	104,029 (92.7%)	51,453 (91.0%)	24,690 (92.5%)	27,886 (96.1%)
Provided home care treatment within the hospital	1180 (1.1%)	635 (1.1%)	330 (1.2%)	215 (0.7%)
Provided home care treatment within another hospital	5074 (4.5%)	3244 (5.7%)	1214 (4.6%)	616 (2.1%)
Unknown	1771 (1.6%)	1125 (2.0%)	386 (1.5%)	260 (0.9%)
Patients receiving treatment after index hospitalization	*n* = 23,659	*n* = 9,391	*n* = 9,240	*n* = 5,028
Persistent to the index treatment regimen, *n* (%)				
No	15,109 (63.9%)	6047 (64.4%)	6011 (65.1%)	3051 (60.7%)
Yes	8550 (36.1%)	3344 (35.6%)	3229 (34.9%)	1977 (39.3%)
MPR for any medication				
Mean (SD), median	0.4 (0.4), 0.3	0.4 (0.4), 0.2	0.4 (0.4), 0.3	0.5 (0.4), 0.3
Range (min, max)	0.0–1.0	0.0–1.0	0.0–1.0	0.0–1.0
MPR category, *n* (%)				
0.00–0.10	8593 (36.3%)	3460 (36.8%)	3347 (36.2%)	1786 (35.5%)
> 0.10–0.20	2409 (10.2%)	1036 (11.0%)	927 (10.0%)	446 (8.9%)
> 0.20–0.30	1799 (7.6%)	690 (7.4%)	742 (8.0%)	367 (7.3%)
> 0.30–0.40	1114 (4.7%)	436 (4.6%)	425 (4.6%)	253 (5.0%)
> 0.40–0.50	714 (3.0%)	305 (3.3%)	276 (3.0%)	133 (2.7%)
> 0.50–0.60	576 (2.4%)	236 (2.5%)	217 (2.4%)	123 (2.5%)
> 0.60–0.70	506 (2.1%)	202 (2.2%)	190 (2.1%)	114 (2.3%)
> 0.70–0.80	300 (1.3%)	97 (1.0%)	137 (1.5%)	66 (1.3%)
> 0.80–0.90	365 (1.5%)	143 (1.5%)	143 (1.6%)	79 (1.6%)
> 0.90–1.00	7283 (30.8%)	2786 (29.7%)	2836 (30.7%)	1661 (33.0%)
Adherent to any osteoporosis treatment, *n* (%)				
No	16,000 (67.6%)	6456 (68.8%)	6258 (67.7%)	3286 (65.4%)
Yes^a^	7659 (32.4%)	2935 (31.3%)	2982 (32.3%)	1742 (34.7%)

*Max*, maximum; *min*, minimum; *MPR*, medication possession ratio; *NVNH*, non-vertebral non-hip; *SD*, standard deviation

^a^Includes 11 patients with an MPR of 0.80

**Table 4:** Treatment regimens from baseline to follow-up after discharge

Medication	Baseline overall (*n* = 9356), *n* (%)	Index hospital visit overall (*n* = 28,678), *n* (%)	Follow-up overall (*n* = 23,659), *n* (%)
Calcium	460 (4.9)	1338 (4.7)	957 (4.0)
Calcium L-aspartate hydrate	460 (100.0)	1334 (99.7)	955 (99.8)
Dibasic calcium phosphate hydrate	0 (0.0)	4 (0.3)	2 (0.2)
Estrogen	266 (2.8)	160 (0.6)	262 (1.1)
Estriol	259 (97.4)	135 (84.4)	251 (95.8)
Estradiol	6 (2.3)	14 (8.8)	8 (3.1)
Conjugated estrogen	2 (0.8)	11 (6.9)	3 (1.2)
Active vitamin D_3_	4778 (51.1)	17,074 (59.5)	13,418 (56.7)
Alfacalcidol	2356 (49.3)	8610 (50.4)	4943 (36.8)
Calcitriol	314 (6.6)	669 (3.9)	363 (2.7)
Eldecalcitol	2263 (47.4)	8538 (50.0)	8630 (64.3)
Bisphosphonate	4441 (47.5)	10,007 (34.9)	11,978 (50.6)
Etidronate disodium	5 (0.1)	3 (0.0)	11 (0.1)
Alendronate sodium hydrate	1873 (42.2)	4875 (48.7)	4509 (37.6)
Clodronate	0 (0.0)	0 (0.0)	0 (0.0)
Sodium risedronate hydrate	1308 (29.5)	3249 (32.5)	3477 (29.0)
Minodronic acid hydrate	1073 (24.2)	1675 (16.7)	3237 (27.0)
Ibandronate	249 (5.6)	408 (4.1)	928 (7.8)
Zoledronic acid	85 (1.9)	118 (1.2)	501 (4.2)
SERM	588 (6.3)	1401 (4.9)	985 (4.2)
Raloxifene hydrochloride	588 (100.0)	1401 (100.0)	985 (100.0)
Bazedoxifene acetate	0 (0.0)	0 (0.0)	0 (0.0)
Calcitonin	471 (5.0)	2706 (9.4)	604 (2.6)
Elcatonin	471 (100.0)	2706 (100.0)	604 (100.0)
Teriparatide	931 (10.0)	4561 (15.9)	3963 (16.8)
Teriparatide acetate^1^	324 (34.8)	1439 (31.6)	1132 (28.6)
Teriparatide (genetical recombination)^1^	626 (67.2)	3196 (70.1)	2899 (73.2)
Denosumab	533 (5.7)	355 (1.2)	2165 (9.2)
Ipriflavone	9 (0.1)	20 (0.1)	9 (0.04)
Nandrolone decanoate	0 (0.0)	0 (0.0)	0 (0.00)
Vitamin K2 (menatetrenone)	584 (6.2)	1408 (4.9)	821 (3.5)
Top first observed treatment regimens			
Bisphosphonate	2431 (26.0)	4714 (4.2)	5977 (25.3)
Active vitamin D_3_	2161 (23.1)	9243 (8.2)	5786 (24.5)
Active vitamin D_3_ + Bisphosphonate	1318 (14.1)	3712 (3.3)	4292 (18.1)
Teriparatide	574 (6.1)	2898 (2.6)	2958 (12.5)
Vitamin K	333 (3.6)	832 (0.7)	423 (1.8)
SERM	247 (2.6)	606 (0.5)	346 (1.5)
Active vitamin D_3_ + SERM	198 (2.1)	−	452 (1.9)
Active vitamin D_3_ + Teriparatide	−	682 (0.6)	373 (1.6)
Active vitamin D_3_ + Calcitonin	−	631 (0.6)	−
Active vitamin D_3_ + Calcium		585 (0.5)	

*SERM*, selective estrogen receptor modulator; − , not applicable

^1^The subcategories “Teriparatide acetate” and “Teriparatide (genetical recombination)” include patients treated with both forms of teriparatide; thus, the sum of both subcategories is greater than 100%

Note: treatment group percentages are calculated out of the total number of treated patients. Subgroup percentages are calculated as a percentage of the associated treatment group

#### Follow-up

Upon discharge, 41.5% (*n* = 46,536) of patients returned to their home, with check-ups scheduled to occur in the same hospital where they were admitted; 34.3% (*n* = 38,542) of patients were transferred to a different hospital or to a medical care facility (Table 3). After discharge, 21.1% (*n* = 23,659; 16.6% hip fracture, 34.6% vertebrae fracture, and 17.3% NVNH fractures) of patients received treatment with antiosteoporotic medications (Table 3). Similar to treatment regimens given during the index hospital visit, the three most common medications administered were active vitamin D_3_ (56.7%, *n* = 13,418), bisphosphonates (50.6%, *n* = 11,978), and teriparatide (16.8%, *n* = 3963).

Treatment initiation for all patients who received treatment after discharge began at a mean of 17.2 days (median, 1 day) after hospital discharge (Table 3). The duration of the first treatment regimen lasted for a mean of 125.7 days (median, 69 days). Patients who discontinued all treatment remained on their initial treatment regimen for a mean 60.3 days (median, 31 days) prior to discontinuing (Table 5). Among the total sample of patients who discontinued (*n* = 15,109), 43.6% (*n* = 6593) of patients either discontinued one regimen and then switched to another or discontinued a regimen and then restarted the same regimen at a later date (Table 5). Patients who discontinued treatment without switching discontinued treatment at a mean of 60.3 days (median, 31 days) following treatment initiation. The mean number of days from initiation of the first treatment to the switch or restart of the second treatment was 141.0 days (median, 127 days). There was a mean of 85 days (median, 80 days) between discontinuing one treatment and switching to or restarting another.

**Table 5:** Treatment patterns for restarting or switching regimens

Characteristic	Overall (*n* = 23,659)	Hip fracture (*n* = 9391)	Vertebrae fracture (*n* = 9240)	NVNH fracture (*n* = 5028)
Duration of treatment for the first observed treatment regimen, d				
Mean (SD), median	125.7 (126.3), 69.0	115.3 (122.2), 57.0	132.3 (128.4), 78.0	133.0 (128.6), 84.0
Range (min, max)	1–365	1–365	1–365	1–365
Patients with treatment discontinuation, *n* (%)	15,109 (63.9)	6047 (64.4)	6011 (65.1)	3051 (60.7)
No. of days from treatment initiation to discontinuation				
Mean (SD), median	58.8 (66.2), 32.0	52.2 (62.0), 29.0	63.3 (67.9), 36.0	62.8 (69.7), 35.0
Range (min, max)	1–302	1–301	1–293	1–302
Patients with discontinuation and no switching/restarting, *n* (%)	8516 (36.0)	3602 (38.4)	3194 (34.6)	1720 (34.2)
No. of days from treatment initiation to discontinuation				
Mean (SD), median	60.3 (68.2), 31.0	54.9 (64.6), 29.0	64.3 (69.3), 36.0	64.4 (72.4), 33.0
Range (min, max)	1–302	1–301	1–293	1–302
Patients with treatment switching/restarting, *n* (%)	6593 (43.6)	2445 (40.4)	2817 (46.9)	1331 (43.6)
No. of days from treatment initiation to treatment switching/restarting				
Mean (SD), median	141.0 (75.3), 127.0	136.7 (69.8), 122.0	141.2 (79.4), 128.0	148.5 (75.6), 141.0
Range (min, max)	16–360	16–356	16–360	16–359
No. of days from treatment discontinuation to treatment switching/restarting				
Mean (SD), median	85.2 (58.7), 80.0	89.3 (57.8), 83.0	80.0 (58.8), 76.0	88.8 (59.1), 80.0
Range (min, max)	2–357	2–356	2–341	2–357
Medication switched to/restarted, *n* (%)				
Calcium	116 (1.8)	28 (1.2)	66 (2.3)	22 (1.7)
Estrogen	100 (1.5)	28 (1.2)	32 (1.1)	40 (3.0)
Active vitamin D_3_	1631 (24.7)	581 (23.8)	718 (25.5)	332 (24.9)
Vitamin K2 (Menatetrenone)	120 (1.8)	51 (2.1)	47 (1.7)	22 (1.7)
Bisphosphonate	3354 (50.9)	1362 (55.7)	1323 (47.0)	669 (50.3)
SERM	114 (1.7)	35 (1.4)	37 (1.3)	42 (3.2)
Calcitonin	104 (1.6)	25 (1.0)	66 (2.3)	13 (1.0)
Teriparatide	1293 (19.6)	332 (13.6)	745 (26.5)	216 (16.2)
Other drugs^a^	850 (12.9)	351 (14.4)	322 (11.4)	177 (13.3)
Patients with treatment augmentation, *n* (%)	1177 (5.0)	400 (4.3)	520 (5.6)	257 (5.1)
No. of days from treatment initiation to treatment augmentation				
Mean (SD), median	116.7 (94.5), 85.0	110.3 (92.4), 76.0	120.2 (96.0), 85.0	119.5 (94.7), 90.0
Range (min, max)	16–360	16–360	17–351	16–353
Augmentation medication, *n* (%)				
Calcium	114 (9.7)	33 (8.3)	55 (10.6)	26 (10.1)
Estrogen	20 (1.7)	14 (3.5)	5 (1.0)	1 (0.4)
Active vitamin D_3_	936 (79.5)	316 (79.0)	414 (79.6)	206 (80.2)
Vitamin K2 (Menatetrenone)	51 (4.3)	13 (3.3)	23 (4.4)	15 (5.8)
Bisphosphonate	536 (45.5)	190 (47.5)	241 (46.4)	105 (40.9)
SERM	57 (4.8)	18 (4.5)	25 (4.8)	14 (5.5)
Calcitonin	57 (4.8)	12 (3.0)	36 (6.9)	9 (3.5)
Teriparatide	142 (12.1)	30 (7.5)	82 (15.8)	30 (11.7)
Other drugs^a^	342 (29.1)	120 (30.0)	136 (26.2)	86 (33.5)

*Max*, maximum; *min*, minimum; *NVNH*, non-vertebral non-hip; *SD*, standard deviation; *SERM,* selective estrogen receptor modulator

^a^Includes denosumab, ipriflavone, and nandrolone

Among patients who switched or restarted treatments (*n* = 6593), patients most frequently switched to bisphosphonates (50.9%, *n* = 3354), active vitamin D_3_ (24.7%, *n* = 1631), teriparatide (19.6%, *n* = 1293), and other medications (12.9%, *n* = 850). Among patients in the fracture cohorts who switched or restarted treatment, 55.7% (*n* = 1362) of patients with hip fracture, 47.0% (*n* = 1323) of patients with vertebrae facture, and 50.3% (*n* = 669) of patients with NVNH fractures switched to a bisphosphonate; 23.8% (*n* = 581) of patients with hip fracture received active vitamin D_3_, as did 25.5% (*n* = 718) of patients with vertebrae fracture, and 24.9% (*n* = 332) of patients with NVNH fractures. Among patients with fractures who switched/restarted, 13.6% (*n* = 332) of patients with hip fracture, 26.5% (*n* = 745) of patients with vertebrae fracture, and 16.2% (*n* = 216) of patients with NVNH fractures switched to or restarted treatment with a teriparatide. Additional details describing the specific switching/restarting treatment regimens can be found in Online Resource 1, Table S-3. A small subset of patients (5.0%, *n* = 1177) augmented treatment, and there was an average of 117 days (median, 85 days) between treatment initiation and augmentation. The most common antiosteoporotic regimens used for augmentation treatment were active vitamin D_3_ (79.5%, *n* = 936), bisphosphonates (45.5%, *n* = 536), and other medications (i.e., ipriflavone, nandrolone decanoate, denosumab) (29.1%, *n* = 342).

#### Treatment adherence

Among patients who received treatment after the index hospitalization (*n* = 23,659), 63.9% (*n* = 15,109) of patients were not adherent to the initial treatment regimen (Table 3). Additionally, 32.4% of patients who received treatment after the index hospitalization had an MPR of ≥ 0.80 (*n* = 7659). Low adherence rates were also observed when we examined the association of adherence rates with other variables, such as concomitant medication at discharge (i.e., patients with and without concomitant medication) and discharge destination (e.g., home, nursing home, geriatric, or social welfare facility). We found that medication adherence was lower in patients with no concomitant medication at discharge (MPR ≥ 0.8 in 28.9% of patients) compared with patients that had concomitant medication at discharge (MPR ≥ 0.8 in 37.8% of patients). We also observed lower adherence in patients discharged to a nursing home, geriatric facility, or social welfare (MPR ≥ 0.8 in 24.9% of patients) compared with patients discharged to their homes (MPR ≥ 0.8 in 36.1% of patients). Finally, similar MPRs were indicated across the three fracture cohorts, with a mean MPR of 0.4 (SD, 0.4) for patients with hip fracture, 0.4 (SD, 0.4) for patients with vertebrae fracture, and 0.5 (SD, 0.4) for patients with NVNH fractures.

#### Treatment receipt at follow-up

Additional variables were examined for their association with receipt of treatment after the index hospitalization visit using multivariable logistic regression (Table 6; area under the curve = 0.7599; 95% CI, 0.7564–0.7634). The odds of receiving treatment upon discharge were 2.11 (95% CI, 1.9–2.3) times higher in older patients aged 70 to 79 years compared with patients aged 50 to 59 years. Furthermore, women had 1.77 (95% CI, 1.7–1.8) times greater odds of receiving treatment than men. Patients had 2.43 (95% CI, 2.3–2.6) times higher odds of receiving treatment if they had a previous osteoporosis diagnosis and 3.23 (95% CI, 2.8–3.8) times higher odds if they had both a previous osteoporosis and a previous fracture diagnosis compared with patients without either of these diagnoses. The odds of receiving medication were 1.71 (95% CI, 1.6–1.8) times greater in patients with a CCI score of ≥ 3 than those with CCI scores of 0. Patients who were discharged to their homes had 2.39 (95% CI, 2.2–2.6) times higher odds of receiving treatment than patients who entered a nursing home, geriatric care facility, or social welfare center. Lastly, patients who were taking ≥ 3 medications at the time of discharge had 6.29 (95% CI, 4.9–8.1) times greater odds of receiving treatment than those who received 0 medications at discharge.

**Table 6:** Multivariable logistic regression analysis of treatment receipt at follow-up

Variable	Odds ratio	95% Confidence interval	*P* value
Age (vs. aged 50–59 *y*), *y*			
60–69	1.79	1.63–1.98	< 0.001
70–79	2.11	1.92–2.32	< 0.001
80–89	1.80	1.64–1.97	< 0.001
≥ 90	1.14	1.03–1.25	0.013
Female (vs. male)	1.77	1.70–1.84	< 0.001
Length of stay for the index hospitalization (vs. < 15 d),^a^ d			
15–30	1.33	1.27–1.39	< 0.001
> 30	1.30	1.24–1.35	< 0.001
Previous hospitalization (vs. no previous hospitalization)	1.30	1.25–1.36	< 0.001
Previous osteoporosis diagnosis/fracture diagnosis (vs. no previous osteoporosis or fracture diagnosis)			
Osteoporosis diagnosis, no fracture diagnosis	2.43	2.28–2.60	< 0.001
Osteoporosis diagnosis and fracture diagnosis	3.23	2.75–3.78	< 0.001
Other route of hospitalization for the index hospitalization (vs. hospitalized from home)	0.66	0.62–0.70	< 0.001
Type of hospitalization for the index hospitalization (vs. planned hospitalization)			
Unplanned hospitalization, nonemergency	1.47	1.40–1.54	< 0.001
Emergency hospitalization	1.44	1.37–1.52	< 0.001
CCI score in the baseline period (vs. 0)			
1	1.38	1.30–1.46	< 0.001
2	1.50	1.41–1.59	< 0.001
3	1.56	1.45–1.68	< 0.001
> 3	1.71	1.62–1.81	< 0.001
Discharge destination (vs. entry in nursing home, geriatric care, social welfare)			
Transferred to same or different hospital	1.02	0.95–1.09	0.620
Discharged home	2.39	2.24–2.55	< 0.001
Other/missing	0.89	0.74–1.07	0.224
No. of medications at discharge (vs. 0)			
1	3.78	3.63–3.94	< 0.001
2	5.11	4.77–5.47	< 0.001
≥ 3	6.29	4.91–8.07	< 0.001

*CCI*, Charlson Comorbidity Index

^a^Categories were determined based on a review of the data

## Discussion

This large retrospective review of patient medical records was conducted to better understand the current treatment patterns of patients hospitalized for osteoporotic fracture in Japan along with the variables associated with receipt of treatment during hospitalization and after discharge. We found that hip fracture was the most common type of hospitalized fracture among both men and women. Most patients who were hospitalized for an osteoporotic fracture initiated their index hospital visit in an unplanned fashion and did not have a previous diagnosis of osteoporosis or a previous fracture. The 0.72% of patients in our study with a previous osteoporotic fracture diagnosis is substantially lower than the 41.5% prevalence rate of multiple fractures in patients with osteoporosis and aged ≥ 50 years that was reported in the 2012–2014 Japan National Health and Wellness Survey [19]. The reason for the discrepancy in rates is unclear but could be partially due to the use of a medical claims database which allowed for patient follow-up only at a single institution (rather than across care settings) versus patient self-report in a survey. Over one-half of the patients in each fracture cohort could perform ADLs, such as eating and transferring, independently or with minor assistance, but patients with hip fracture needed more assistance with grooming, using the toilet, and walking at ground level or on stairs, and patients from all three cohorts needed assistance with bathing.

Most patients were not taking antiosteoporotic medications prior to their index hospital visit; and while in the hospital, 25.5% of the total patient sample received antiosteoporotic medications for treatment of their fracture. The most common treatment regimens for fracture were active vitamin D_3_, bisphosphonates, and teriparatide, and these medications were sometimes given in combination. Calcitonin was another medication prescribed for pain relief from fracture during the hospital visit and to a lesser extent upon discharge. The treatment patterns observed in this study are broadly aligned with the results of other studies conducted across different countries (United States [20, 21]; China, Hong Kong, Singapore, South Korea, Malaysia, Taiwan, and Thailand [22]; Korea [21]; Spain [21]; and the UK [23]), which found that bisphosphonates were the most commonly used drug category after osteoporotic fracture. Additionally, studies conducted in India [24], Italy [25], and France [26] observed that vitamin D_3_ was frequently prescribed to patients after fracture, although the literature regarding vitamin D_3_ supplementation patterns post-fracture is limited. Moreover, in agreement with our findings, bisphosphonates and active vitamin D_3_ were the most commonly prescribed treatments after hip and vertebral fracture in a recent analysis of health insurance claims in Japan [27]. In the current study, approximately 21% of the total patient sample received treatment with antiosteoporotic medications upon discharge, which was similar to the rate of 18.7% observed in a previous chart review study of female patients treated at 25 hospitals in Japan [6]. In our analysis, we found that several variables contributed to the likelihood of patients receiving treatment after discharge: patients who were older than 59 years, who were female, who had higher CCI scores, and who received three or more medications upon discharge were more likely to receive treatment after discharge. Although treatment rate was higher in patients who were discharged to their home rather than to a nursing home or geriatric care center, adherence was generally low, regardless of discharge destination.

The literature regarding osteoporosis treatment patterns and adherence outcomes suggests insufficient adherence to antiosteoporotic medications [18, 28, 29]. In the current study, we found that only 32.4% of patients who received treatment after the index hospitalization were adherent to their medication (MPR ≥ 0.80) during the 12-month observation period. In contrast to our findings, Nakatoh et al. [18] observed higher medication adherence over a 2-year follow-up period in Japanese patients with osteoporosis (MPR ≥ 0.80 in 49.6% of patients), although the authors concluded that these adherence rates remained inadequate. Similar to our observation that medication adherence is higher in patients with concomitant medication at discharge, Nakatoh et al. [18] found that medication adherence during the 2-year follow-up period was moderately higher in patients with polypharmacy compared to patients without polypharmacy. A population-based cohort study in Taiwan [29] examined the association of adherence to antiosteoporotic agents with mortality risk in older adults with hip fractures, using data from the Taiwan National Health Insurance Database. Results showed that individuals with higher medication adherence rates had lower mortality rates at 1, 3, and 5 years following the hip fracture than individuals with lower treatment adherence [29]. These results underscore the importance of treatment adherence in achieving longer-term benefits. Furthermore, a systematic literature review in 2019 was conducted to understand the condition-related factors for nonadherence with antiosteoporotic medications, including motivation of patients, side effects of medication, perceived benefits, and forgetfulness [28]. The authors acknowledged the challenge of low treatment compliance and offered solutions for improving adherence, including patient education, dosing simplification, and flexibility [28]. Overall, treatment with antiosteoporotic medications can improve health outcomes, yet adherence is a common challenge [28, 29].

One limitation of this study is the possibility that patients had osteoporosis treatment for several years prior to the captured baseline period for the current study. Because we had 1 year of baseline data available on which to base results, the current results may overestimate the rate of patients who did not have prior osteoporosis treatment. Another limitation was that only data within the medical records were available for analysis, as there was no communication with patients or healthcare providers to seek additional information; therefore, results may not be generalizable to all patients who were hospitalized for osteoporotic fracture. For example, although approximately 50% of the patients were discharged to their home, another 34% were discharged to another hospital or medical facility. After discharge to another facility, it may be possible that these patients received additional nonpharmacological care such as physical therapy, and hence the patients did not adhere to the initial regimen. Furthermore, the use of a hospital-based database allowed for patient follow-up only at a single institution, which may have limited the information available for patients that sought care across multiple settings. Use of the MDV database may also limit generalizability to the general Japanese population, as it contains data for hospitalized patients and outpatients in need of healthcare services. However, the database is suitable for our population of interest, as fracture patients in Japan are typically referred to acute-care hospitals [30]. A strength was the large sample size of available patient data for abstraction and analysis. The MDV database contained much relevant data for examining treatment patterns from multiple hospitals in Japan and allowed for a longitudinal assessment of variables. Furthermore, the retrospective design of this study allowed for the examination of real-world patient data, which supports generalizability of results.

## Conclusion

This study provides an updated treatment landscape for clinical characteristics and treatment patterns of older individuals in Japan who were hospitalized for treatment of osteoporotic fracture. Despite osteoporotic fracture being a major health concern in older Japanese populations, both treatment with antiosteoporotic medications and adherence to treatment regimens remain generally low.

### Supplementary Information

Below is the link to the electronic supplementary material.

Supplementary file1 (PDF 243 KB)

## Data Availability

The data are not publicly available.
