# Supplementary material for: A real-world study of treatment patterns among patients with osteoporotic fracture: analysis of a Japanese hospital database
Source: Arch Osteoporos. 2023 Jan 23;18(1):23. doi: 10.1007/s11657-022-01201-x (PMC9868038; doi:10.1007/s11657-022-01201-x)
Supplement: Supplementary file 1 — Supplementary file1 (PDF 243 KB) [file 11657_2022_1201_MOESM1_ESM.pdf]

FIGURES

Fig. S-1 Study Timeline

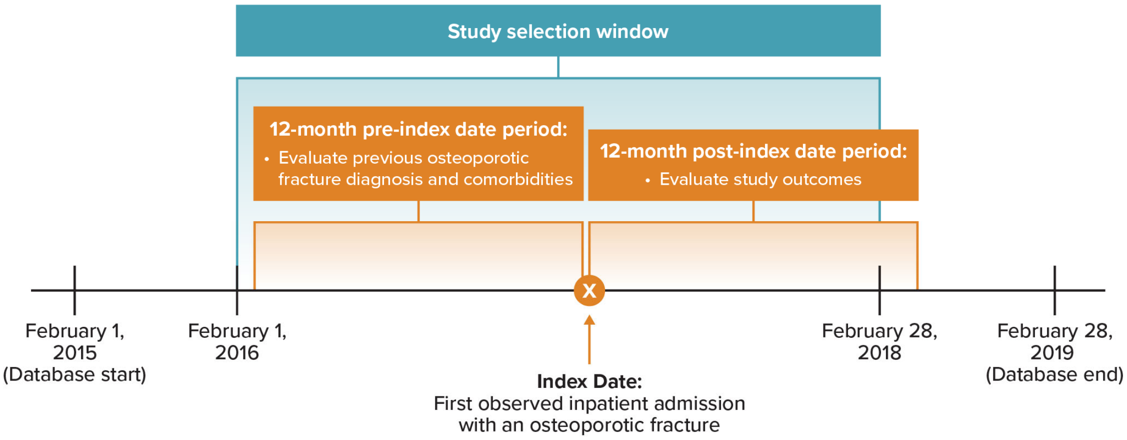

Fig. S-2 Process of Patient Selection

Patients with a diagnosis of osteoporotic fracture[1] on an inpatient admission between February 1, 2016 and February 28, 2018 (date of admission for the first observed hospitalization with a diagnosis of osteoporotic fracture termed the index date)

N = 137,722

↓

Patients aged at least 50 years on the index date

N = 137,722 (100%)[2]

↓

Patients with a single fracture site on their index hospitalization

N = 137,410 (99.8%)[2]

↓

Patients with at least 12 months of data available after before admission to index hospital admission

N = 117,674 (85.4%)[2]

↓

Patients with at least 12 months of data available after discharge from their index hospital admission

N = 112,567 (81.7%)[2]

↓

Patients with > 0 length of stay for their index hospital admission

N = 112,275 (81.5%)[2]

[1] Osteoporotic fracture diagnosis based on discharge diagnosis data.

[2] Percentage of the initial cohort of patients with a diagnosis of osteoporotic fracture on an inpatient admission between February 1, 2016 and February 28, 2018.

TABLES

Table S-1. ICD-10 Codes to Identify Osteoporotic Fractures

ICD-10 Code	Fracture Category	ICD-10 Name
S7200	Hip	Fracture of neck of femur
S7201	Hip	Fracture of neck of femur
S7210	Hip	Pertrochanteric fracture
S7211	Hip	Pertrochanteric fracture
S7220	Hip	Subtrochanteric fracture
S7221	Hip	Subtrochanteric fracture
S2230	Non-vertebral excluding hip	Fracture of rib
S2231	Non-vertebral excluding hip	Fracture of rib
S2240	Non-vertebral excluding hip	Multiple fractures of ribs
S2241	Non-vertebral excluding hip	Multiple fractures of ribs
S3210	Non-vertebral excluding hip	Fracture of sacrum
S3211	Non-vertebral excluding hip	Fracture of sacrum
S3250	Non-vertebral excluding hip	Fracture of pubis
S3251	Non-vertebral excluding hip	Fracture of pubis
S4220	Non-vertebral excluding hip	Fracture of upper end of humerus
S4221	Non-vertebral excluding hip	Fracture of upper end of humerus
S5250	Non-vertebral excluding hip	Fracture of lower end of radius
S5251	Non-vertebral excluding hip	Fracture of lower end of radius
S5260	Non-vertebral excluding hip	Fracture of lower end of both ulna and radius
S5261	Non-vertebral excluding hip	Fracture of lower end of both ulna and radius
S5290	Non-vertebral excluding hip	Fracture of forearm, part unspecified
S5291	Non-vertebral excluding hip	Fracture of forearm, part unspecified
S7290	Non-vertebral excluding hip	Fracture of femur, part unspecified
S7291	Non-vertebral excluding hip	Fracture of femur, part unspecified
S8210	Non-vertebral excluding hip	Fracture of upper end of tibia
S8211	Non-vertebral excluding hip	Fracture of upper end of tibia
S8230	Non-vertebral excluding hip	Fracture of lower end of tibia
S8231	Non-vertebral excluding hip	Fracture of lower end of tibia
S8240	Non-vertebral excluding hip	Fracture of fibula alone
S8241	Non-vertebral excluding hip	Fracture of fibula alone
T08	Non-vertebral excluding hip	Fracture of spine, level unspecified

ICD-10 Code	Fracture Category	ICD-10 Name
S2200	Vertebral	Fracture of thoracic vertebra
S2201	Vertebral	Fracture of thoracic vertebra
S2210	Vertebral	Multiple fractures of thoracic spine
S2211	Vertebral	Multiple fractures of thoracic spine
S320	Vertebral	Fracture of lumbar vertebra
S320	Vertebral	Fracture of lumbar vertebra
S3200	Vertebral	Fracture of lumbar vertebra
S3200	Vertebral	Fracture of lumbar vertebra
S3201	Vertebral	Fracture of lumbar vertebra
S3201	Vertebral	Fracture of lumbar vertebra

Table S-2. Patient Comorbid Conditions

Characteristics	Overall	Hip Fracture	Vertebrae Fracture	NVNH Fracture
Charlson comorbidities[a,b] during baseline (n, %)				
Cancer, nonmetastatic	12,180 (10.9%)	5,388 (9.5%)	3,858 (14.5%)	2,934 (10.1%)
Hypertension	10,458 (9.3%)	4,876 (8.6%)	3,067 (11.5%)	2,515 (8.7%)
Congestive heart failure	10,194 (9.1%)	4,654 (8.2%)	2,908 (10.9%)	2,632 (9.1%)
Cerebrovascular disease	9,294 (8.3%)	4,593 (8.1%)	2,777 (10.4%)	1,924 (6.6%)
Mild liver disease	6,623 (5.9%)	2,619 (4.6%)	1,951 (7.3%)	2,053 (7.1%)
Chronic pulmonary disease	4,678 (4.2%)	1,853 (3.3%)	1,495 (5.6%)	1,330 (4.6%)
Ulcer disease	4,258 (3.8%)	1,872 (3.3%)	1,257 (4.7%)	1,129 (3.9%)
Diabetes without complications	3,895 (3.5%)	1,644 (2.9%)	1,003 (3.8%)	1,248 (4.3%)
Myocardial infarction	3,497 (3.1%)	1,694 (3.0%)	993 (3.7%)	810 (2.8%)
Dementia	3,218 (2.9%)	2,023 (3.6%)	745 (2.8%)	450 (1.6%)
CCI score[c]				
Mean (SD), median	1.1 (2.3), 0.0	1.0 (2.2), 0.0	1.4 (2.5), 0.0	1.0 (2.1), 0.0
Range (min, max)	0-22	0-22	0-22	0-22
Charlson comorbidities at index hospital visit (n, %)				
Hypertension	43,795 (39.0%)	24,185 (42.8%)	10,580 (39.7%)	9,030 (31.1%)
Dementia	16,691 (14.9%)	12,466 (22.0%)	2,584 (9.7%)	1,641 (5.7%)
Diabetes without complications	15,667 (14.0%)	8,512 (15.1%)	3,476 (13.0%)	3,679 (12.7%)
Cerebrovascular disease	13,290 (11.8%)	8,147 (14.4%)	3,187 (11.9%)	1,956 (6.7%)

Characteristics	Overall	Hip Fracture	Vertebrae Fracture	NVNH Fracture
Congestive heart failure	11,278 (10.0%)	7,177 (12.7%)	2,575 (9.7%)	1,526 (5.3%)
Cancer, nonmetastatic	7,920 (7.1%)	4,203 (7.4%)	2,472 (9.3%)	1,245 (4.3%)
Ulcer disease	7,501 (6.7%)	3,889 (6.9%)	1,855 (7.0%)	1,757 (6.1%)
Chronic pulmonary disease	6,193 (5.5%)	3,355 (5.9%)	1,712 (6.4%)	1,126 (3.9%)
Renal disease	5,394 (4.8%)	3,462 (6.1%)	1,111 (4.2%)	821 (2.8%)
Mild liver disease	5,089 (4.5%)	2,887 (5.1%)	1,108 (4.2%)	1,094 (3.8%)
CCI score				
Mean (SD), median	1.6 (1.6), 1.0	1.8 (1.7), 2.0	1.6 (1.7), 1.0	1.1 (1.4), 1.0
Range (min, max)	0-17	0-17	0-14	0-13
Other comorbidities during baseline (n, %)				
Cerebrovascular disease	10,452 (9.3%)	6,587 (11.6%)	2,395 (9.0%)	1,470 (5.1%)
Congestive heart failure	8,454 (7.5%)	5,621 (9.9%)	1,747 (6.6%)	1,086 (3.7%)
Peripheral vascular disease	1,616 (1.4%)	950 (1.7%)	431 (1.6%)	235 (0.8%)
Myocardial infarction	1,510 (1.3%)	940 (1.7%)	333 (1.3%)	237 (0.8%)

CCI, Charlson Comorbidity Index; max, maximum; min, minimum, n, number of patients in a group/condition; NVNH, non-vertebral non-hip; SD, standard deviation.

[a] Ten most prevalent CCI comorbidities.

[b] Additional Charlson comorbidities included peripheral vascular disease, renal disease, cancer – metastatic, rheumatic disease, diabetes with complications, skin ulcers, depression, HIV/AIDS, moderate-to-severe liver disease, and hemiplegia or paraplegia.

[c] Includes all CCI comorbidities.

Table S-3. Switching/Restarting Medication Regimens for All Patients

Treatment Regimen[a]	Bisphosphonate (n = 2,777)	Active Vitamin D_3 (n = 750)	Active Vitamin D_3 + Bisphosphonate (n = 442)	Teriparatide (n = 1,408)	Other[b] (n = 543)	Active Vitamin D_3 + Other (n = 77)	Active Vitamin D_3 + SERM (n = 51)	Vitamin K (n = 49)	Active Vitamin D_3 + Teriparatide (n = 72)	SERM (n = 52)
Switched/ restarted regimen										
Bisphosphonate	2,311 (83.2%)	73 (9.7%)	100 (22.6%)	149 (10.6%)	8 (1.5%)	2 (2.6%)	3 (5.9%)	10 (20.4%)	12 (16.7%)	7 (13.5%)
Teriparatide	91 (3.3%)	54 (7.2%)	42 (9.5%)	871 (61.9%)	1 (0.2%)	3 (3.9%)	9 (17.7%)	3 (6.1%)	23 (31.9%)	5 (9.6%)
Other[b]	36 (1.3%)	43 (5.7%)	27 (6.1%)	63 (4.5%)	467 (86.0%)	44 (57.1%)	1 (2.0%)	1 (2.0%)	3 (4.2%)	1 (1.9%)
Active vitamin D_3	128 (4.6%)	312 (41.6%)	65 (14.7%)	88 (6.3%)	44 (8.1%)	11 (14.3%)	7 (13.7%)	6 (12.2%)	9 (12.5%)	9 (17.3%)
Bisphosphonate + active vitamin D_3	120 (4.3%)	124 (16.5%)	150 (33.9%)	81 (5.8%)	0 (0.0%)	0 (0.0%)	4 (7.8%)	0 (0.0%)	12 (16.7%)	3 (5.8%)
Other[a] + active vitamin D_3	8 (0.3%)	53 (7.1%)	5 (1.1%)	25 (1.8%)	7 (1.3%)	12 (15.6%)	1 (2.0%)	0 (0.0%)	1 (1.4%)	0 (0.0%)
Teriparatide + active vitamin D_3	1 (0.0%)	19 (2.5%)	14 (3.2%)	42 (3.0%)	0 (0.0%)	0 (0.0%)	0 (0.0%)	1 (2.0%)	6 (8.3%)	0 (0.0%)
Vitamin K	18 (0.7%)	10 (1.3%)	8 (1.8%)	3 (0.2%)	2 (0.4%)	1 (1.3%)	4 (7.8%)	20 (40.8%)	1 (1.4%)	1 (1.9%)
SERM	6 (0.2%)	10 (1.3%)	5 (1.1%)	6 (0.4%)	0 (0.0%)	1 (1.3%)	3 (5.9%)	1 (2.0%)	0 (0.0%)	20 (38.5%)
SERM + active vitamin D_3	6 (0.2%)	12 (1.6%)	5 (1.1%)	6 (0.4%)	1 (0.2%)	0 (0.0%)	15 (29.4%)	0 (0.0%)	0 (0.0%)	4 (7.7%)

Treatment Regimen[a]	Bisphosphonate (n = 2,777)	Active Vitamin D_3 (n = 750)	Active Vitamin D_3 + Bisphosphonate (n = 442)	Teriparatide (n = 1,408)	Other[b] (n = 543)	Active Vitamin D_3 + Other (n = 77)	Active Vitamin D_3 + SERM (n = 51)	Vitamin K (n = 49)	Active Vitamin D_3 + Teriparatide (n = 72)	SERM (n = 52)
Calcitonin	17 (0.6%)	16 (2.1%)	2 (0.5%)	3 (0.2%)	4 (0.7%)	0 (0.0%)	0 (0.0%)	2 (4.1%)	0 (0.0%)	1 (1.9%)
Estrogen	8 (0.3%)	3 (0.4%)	5 (1.1%)	6 (0.4%)	1 (0.2%)	0 (0.0%)	1 (2.0%)	0 (0.0%)	2 (2.8%)	0 (0.0%)
Calcium + active vitamin D_3	4 (0.1%)	3 (0.4%)	3 (0.7%)	8 (0.6%)	2 (0.4%)	1 (1.3%)	0 (0.0%)	0 (0.0%)	0 (0.0%)	0 (0.0%)
Bisphosphonate + teriparatide	2 (0.0%)	0 (0.0%)	0 (0.0%)	16 (1.1%)	0 (0.0%)	0 (0.0%)	0 (0.0%)	0 (0.0%)	0 (0.0%)	0 (0.0%)
Bisphosphonate + teriparatide + active vitamin D_3	0 (0.0%)	1 (0.1%)	2 (0.5%)	12 (0.9%)	0 (0.0%)	0 (0.0%)	0 (0.0%)	0 (0.0%)	1 (1.4%)	0 (0.0%)
Bisphosphonate + calcium + active vitamin D_3	3 (0.1%)	1 (0.1%)	0 (0.0%)	10 (0.7%)	0 (0.0%)	1 (1.3%)	0 (0.0%)	0 (0.0%)	0 (0.0%)	0 (0.0%)

SERM, selective estrogen receptor modulators.

[a]Column heading n's represent patients in the starting index treatment regimens.

[b]Ipriflavone, nandrolone decanoate, denosumab.
